# Immune Priming of Pacific Oysters (*Crassostrea gigas*) to Induce Resistance to *Ostreid herpesvirus 1*: Comparison of Infectious and Inactivated OsHV-1 with Poly I:C

**DOI:** 10.3390/v15091943

**Published:** 2023-09-17

**Authors:** Maximilian de Kantzow, Paul M. Hick, Richard J. Whittington

**Affiliations:** School of Veterinary Science, Faculty of Science, The University of Sydney, 425 Werombi Road, Camden, NSW 2570, Australia

**Keywords:** *Ostreid herpesvirus 1* (OsHV-1), Pacific oyster, *Crassostrea gigas*, immunity, resistance, susceptibility, experimental infection, disease control

## Abstract

Pacific oyster mortality syndrome (POMS), which is caused by *Ostreid herpesvirus 1* (OsHV-1), causes economic losses in Pacific oyster (*Crassostrea gigas*) aquaculture in many countries. Reducing the mortality in disease outbreaks requires changing the host, pathogen and environment interactions to favor the host. Survivors of natural exposure to OsHV-1 are able to survive subsequent outbreaks. This has been replicated under laboratory conditions, suggesting the existence of an immune response. The aim of the present study is to compare the effects of prior exposure to infectious OsHV-1, heat-inactivated OsHV-1 and the chemical anti-viral immune stimulant poly I:C on mortality following exposure to virulent OsHV-1. All treatments were administered by intramuscular injection. Oysters were maintained at 18 °C for 14 days; then, the temperature was increased to 22 °C and the oysters were challenged with virulent OsHV-1. Heat-inactivated OsHV-1, infectious OsHV-1 and poly I:C all induced significant protection against mortality, with the hazard of death being 0.41, 0.18 and 0.02, respectively, compared to the controls, which had no immune priming. The replication of OsHV-1 on first exposure was not required to induce a protective response. While the underlying mechanisms for protection remain to be elucidated, conditioning for resistance to POMS by prior exposure to inactivated or infectious OsHV-1 may have practical applications in oyster farming but requires further development to optimize the dose and delivery mechanism and evaluate the duration of protection.

## 1. Introduction

Pacific oysters (*Crassostrea gigas*) are affected by seasonal outbreaks of disease due to diverse genotypes of *Ostreid herpesvirus 1* (OsHV-1) (order of *Herpesvirales*, family *Malacoherpesviridae*) in Europe, Australia and New Zealand, causing substantial economic losses [1,2,3,4,5]. Reducing the impact of OsHV-1 on Pacific oyster aquaculture requires an integrated approach to disease management [6]. Currently, the focus of disease control includes the selection of oyster stocks with genetic resistance [7] and changing the growing conditions, for example, by raising the growing height or filtering the virus from the water to favorably modify the environment [8,9,10,11,12]. Immune priming has also been explored as a way of reducing the level of mortality during an OsHV-1 challenge [13,14,15]. Vaccines are frequently used in finfish aquaculture to protect the host from infection by stimulating specific antibodies against a pathogen, but this approach is not available for invertebrates such as oysters as they do not produce antibodies [16]. However, in crustaceans, the administration of inactivated white spot syndrome virus (WSSV) with the immunostimulant glucan or inactivated *Vibrio penaeicida* achieved a reduction in mortality following a WSSV challenge [17]. The mechanism behind the increased survival is increased phagocytic activity mediated by pattern recognition receptor (PRR) proteins, which bind to pathogen-associated molecular patterns (PAMPs) on the surface of the pathogen [18]. There is evidence that many types of invertebrates have a form of immune memory arising from prior exposure, which reduces mortality in subsequent exposures [19,20]. In *C. gigas*, this was reported for *Vibrio splendidus* bacteria [21] and for OsHV-1 [22].

Immune priming of invertebrates has been defined as a process resulting in greater survival in the second encounter with a pathogen by virtue of a change induced by the first exposure [20,23,24]. A specific immune response was observed in the copepod *Macrocyclops albidus* with an initial exposure to the parasitic tapeworm *Schistocephalus solidus*, resulting in a more effective immune response against the same strain upon a second exposure, but no difference was found in response to a related strain [19]. This indicated the involvement of immune memory, which is the ability to store and reuse information about a previously encountered pathogen. Memory may reside in the form of long-lived RNA-induced silencing complexes (RISCs) incorporating viral gene-specific miRNA, which selectively silence viral gene expression [20,25]. The administration of dsRNA of the VP26 or VP28 WSSV gene or recombinant VP26 or VP28 protein protected *Litopenaeus vannamei* and *Penaeus japonicus*, respectively, against a lethal WSSV challenge [17,26]. Similarly, injecting *C. gigas* with polyinosinic:polycytidylic acid (poly I:C), a synthetic double-stranded homopolymer-mimicking dsRNA, protected oysters against OsHV-1 [13,27,28].

Poly I:C is a potent immune stimulant that binds to conserved pathogen-associated molecular pattern (PAMP) receptors such as Toll-like receptors (TLRs), as it is an analogue of viral dsRNA. Administering poly I:C to oysters prevented mortality from an OsHV-1 challenge up to 18 weeks later [13,14,28,29]. A transgenerational effect was also reported: at 96 h after exposure to OsHV-1, the mortality in the larvae of brood stock treated with poly I:C was halved compared to the larvae of brood stock treated with seawater [13]. The administration of poly I:C changes the gene expression patterns in immune pathways that are presumed to act as antiviral defenses [14,30]. Because poly I:C is not composed of deoxyribonucleotides in meaningful codons, it has nothing uniquely in common with OsHV-1 over other viruses. The administration of two consecutive doses of poly I:C did not induce changes in the transcription of immune-associated genes in the second administration [31]. Importantly, poly I:C provided no protection against infection with the pathogen *Vibrio tasmaniensis*, suggesting that the protective effect may be limited to viruses [14,29].

Field observations suggest that oysters that have previously been exposed to OsHV-1 during an outbreak are likely to survive subsequent outbreaks [32]. They also survive laboratory challenges with virulent OsHV-1 [33]. Beyond the effects of genetic variation, a proportion of the increased survival is due to the increasing age and size, reducing the susceptibility of the oysters between outbreaks [34], but repeated exposure to OSHV-1 per se may also reduce mortality. Under laboratory conditions, OsHV-1 exposure with the water temperature at 18 °C enabled the infection of oysters without high mortality, and this protected them against subsequent OsHV-1 challenges at 22 °C, a water temperature at which high mortality would be expected [22]; note that in Australia, water temperatures that are conducive to mass mortality are several degrees Celsius higher than those reported in Europe [35,36]. It is possible that these observations of protection are due to a general immune stimulation or the disruption of a cellular process required for viral replication, and as yet, there is no experimental evidence that they represent a specific response.

The aim of this experiment was to establish whether active infection with OsHV-1 is required to produce a protective effect or whether exposure to inactivated OsHV-1, which comprises the envelope, capsid, nucleic acid and other molecules, would be sufficient. Conditioning oysters by exposure to heat-inactivated OsHV-1 may allow immune priming, which may indicate underlying mechanisms that are not dependent on viral replication. A specific temperature regime known to lead to protection after exposure to infectious OsHV-1 was used and poly I:C was included as a reference treatment.

## 2. Materials and Methods

### 2.1. The Experimental Design

**Figure 1:**
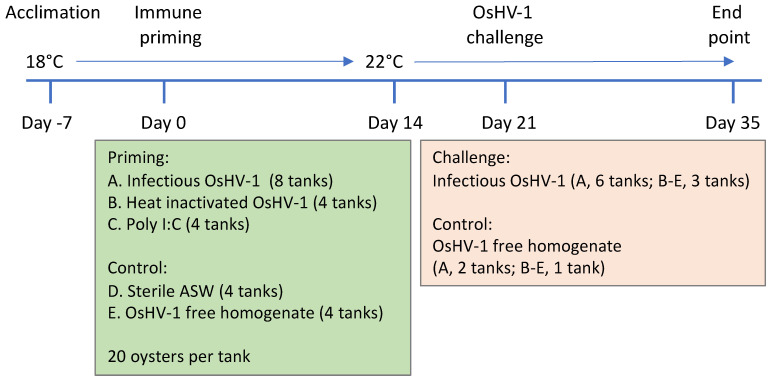
Timeline showing the acclimation period, immune priming and the challenge with OsHV-1. The water temperature was increased from 18 °C to 22 °C on day 14. Samples were taken to measure OsHV-1 prevalence and DNA concentration immediately prior to the OsHV-1 challenge on day 21, in addition to being taken from all dead oysters and at the end of the trial from all surviving oysters.

The experimental design is illustrated in Figure 1. Three immune priming treatments were tested for efficacy in protecting *C. gigas* against mortality when challenged with OsHV-1: A. infectious OsHV-1 (3 × 10^6^ genome copies per oyster), B. heat-inactivated OsHV-1 (3 × 10^6^ genome copies per oyster) and C. poly I:C (250 µg per oyster). The control treatments were D. sterile artificial sea water and E. OsHV-1-free oyster tissue homogenate. There were 80 oysters per treatment group except for in the group primed with infectious OsHV-1, which had 160 to allow for mortality after immune priming. The five treatments were administered by injection and the oysters were maintained in laboratory aquaria with the water temperature at 18 °C for 14 days. On day 14 the temperature was increased to 22 °C over 6 h. One week later (three weeks after immune priming), three quarters of the replicate tanks for each treatment group were randomly selected and the oysters in these were challenged with infectious OsHV-1. Oysters in the remaining tank(s) in each treatment group acted as negative controls and were challenged with OsHV-1-free oyster tissue homogenate. Oysters were monitored for 14 days after the challenge. The same virus variant was used for immune priming and for the challenge.

### 2.2. Oysters

Triploid Pacific oysters (Shellfish Culture Tasmania, Batch SPL17C) were acquired as spat and grown in commercial farming conditions in Patonga Creek, New South Wales (NSW), from March 2018. The oysters were from an OsHV-1-free hatchery that undergoes testing as part of a biosecurity program for OsHV-1 and were batch certified as free from infection with OsHV-1 when transferred from Tasmania to NSW. A random sample of 30 tested negative for OsHV-1 by qPCR immediately prior to the trial in October 2018. The oysters (20–30 mm length) were randomly assigned to tanks and acclimated to laboratory conditions at 18 °C for 7 days (Figure 1).

### 2.3. Aquarium Management

Each tank contained 12 L of artificial sea water (ASW) (Red Sea Salt) at 30 ppt, with a biofilter and an air stone. There were 20 oysters per tank. The oysters were placed on an elevated, perforated polycarbonate rack to allow water circulation and ensure access to feed. The water temperature was maintained by placing the tanks in a water bath; there were 6 tanks in each of the 4 water baths, as previously described [22]. The photoperiod was 12 h of light per day. The oysters were fed a commercial, live algae concentrate (Shellfish Diet 1800, Reed Mariculture) at a rate of 2 mL per tank every second day. Water quality was measured in a subset of tanks prior to feeding and was maintained with a total ammonia nitrogen concentration <2 ppm and pH 8.2–7.8 by water exchange or the addition of sodium bicarbonate as required. A complete water exchange was performed in all tanks every 14 days.

### 2.4. Immune Priming

Each tank was randomly allocated to one of the five treatments using the RAND function in Microsoft Office Excel 365 (Microsoft Corporation, Seattle, WA, USA). All of the oysters in a tank were injected in the adductor muscle with a 50 µL volume of the allotted treatment using a 1 mL syringe and a 25 G needle. The oysters were removed from the water 12 h prior to this and placed in a solution of 50 g·L^−1^ magnesium chloride (Sigma, Castle Hill, NSW, Australia) in fresh aquarium water for 4 h to relax the adductor muscle.

ASW (Red Sea Salt) was prepared at 30 ppt in purified water (MilliQ), filtered to 0.22 µm (Micropore) and used as a negative control for injection and dilution of reagents.

The OsHV-1-free oyster tissue homogenate was prepared from a tissue homogenate of healthy oysters as described below. To achieve a final dilution of 1 in 1000 *v*/*v*, 200 µL of the supernatant was added to 19.8 mL of ASW and filtered to 0.22 µm.

The infectious OsHV-1 treatment was prepared from a cryopreserved OsHV-1 stock (V171) produced from naturally infected oysters sampled from the Georges River on 24 November 2011 (SVC 11/245) [8]; the genome sequence of OsHV-1 in oyster samples collected from this location on the same date has been partially characterized [4]. Briefly, in April 2014, the gill and mantle tissues of 5 whole oysters that had been stored at −80 °C for 29 months were excised and pooled, diluted to 10% *w*/*v* in sterile ASW, homogenized and centrifuged at 1000× *g* for 5 min at 4 °C; then, the supernatant was filtered to 0.22 um and frozen slowly at −80 °C with 10% *v*/*v* fetal bovine serum (Sigma) and 10% *v*/*v* glycerol (Sigma). Immediately before the experiment in October 2018, the cryopreserved V171 stock was thawed and diluted 1 in 1000 with sterile ASW to create the infectious OsHV-1 inoculum.

Heat-inactivated OsHV-1 was prepared by heating 10 mL of the infectious OsHV-1 inoculum to 50 °C for 20 min in a hybridization oven according to Hick et al. [37].

The poly I:C inoculum was prepared by diluting lyophilized poly I:C (Sigma) to 5 mg.mL^−1^ in ultrapure water (Life Technologies, Mulgrave, VIC, Australia). All treatments were prepared immediately prior to use.

### 2.5. OsHV-1 Challenge

After relaxation in 50 g·L^−1^ magnesium chloride in ASW at 22 °C for 6 h, oysters were injected into the adductor muscle with 50 µL of freshly prepared infectious OsHV-1 inoculum diluted to contain 6 × 10^6^ OsHV-1 genome copies per dose, or with the same volume of freshly prepared OsHV-1-free oyster tissue homogenate (negative control inoculum). Oysters were observed every 12 h for 14 days, and dead or moribund oysters were removed and stored at −80 °C.

### 2.6. Quantification of OsHV-1 DNA

A gill and mantle sample (approximately equal portions, total 0.08–0.12 g) from an individual oyster was placed into a 2.0 mL tube containing 1.0 mL of DNase/Rnase-free ultrapure water (Life Technologies) and 0.4 mL of silica zirconia beads (Daintree Scientific, Saint Helens, TAS, Australia). Mechanical homogenization was performed with a TissueLyser II (Qiagen, Clayton, VIC, Australia) for 240 s at 30 oscillations per second, with an inversion of the tube holder after 120 s. The tubes were centrifuged at 900× *g* for 5 min and the supernatant was stored at −80 °C until nucleic acid extraction. Total nucleic acids were purified from 50 µL of supernatant using the MagMAX-96 Viral RNA Isolation Kit (ThermoFisher Scientific, North Ryde, NSW, Australia) and a magnetic particle processor (MagMAX Express-96 Applied Biosystems, ThermoFisher Scientific, North Ryde, NSW, Australia) according to the manufacturer’s directions. Purified nucleic acids were stored at −20 °C prior to quantification of OsHV-1 DNA using the qPCR assay targeting the B-region of the OsHV-1 genome (ORF 99, IAP), as described [38,39]. All samples, controls and standards were tested in duplicate. Quantitative data were converted to OsHV-1 genome copies per mg of oyster tissue.

### 2.7. Statistical Analysis

Statistical analysis was performed in Microsoft R Open version 3.4.5 [40] using the tibble, readr, tidyr, plyr and forcats packages to manage and summarize the data. The sample size was calculated with 80% power and α = 0.05 for an expected hazard ratio between the treatment and control groups of 4 in R 3.4.5 using the powerSurvEpi package. The calculation required an assumption that mortality would be 10% in oysters pre-exposed to OsHV-1 at 18 °C, 50% in oysters pre-exposed to ASW and 20% in control oysters pre-exposed to negative inoculum. The sample size for each group was then rounded up to a multiple of 20 to allow for 20 oysters per replicate tank.

Summary tables of the total cumulative mortality and Kaplan–Meier survival curves were produced using the survminer package to plot the curves. The mortality data were analyzed using a Cox proportional hazards model with the survival package. The failure condition was mortality within the follow-up period after the challenge (14 days) and a positive OsHV-1 PCR result at the time of mortality. The model included treatment group as the only independent variable and tank identification number was included as a random effect to account for clustering within replicate tanks. The Wald test statistic was used to assess the significance of each treatment and the Schoenfeld residuals were plotted to assess the assumption of proportional hazards. Relative percent survival was calculated according to Jarp and Tverdal [41].

OsHV-1 DNA concentration was log_10_ transformed for graphical presentation and to satisfy the assumption of normality. Boxplots were created using ggplot2. Confidence intervals for the mean OsHV-1 concentration from each group were calculated with 95% confidence intervals and back transformed for presentation. The OsHV-1 DNA concentrations at the time of death and in the survivors at the end of the trial were analyzed separately. Both data sets were analyzed using a linear mixed model with the glm function, with log_10_-transformed OsHV-1 concentration as the outcome and treatment group as the explanatory variable; tank number was included as a random effect to account for clustering within replicate tanks. Confidence intervals for the model parameters were calculated at the 95% level.

A summary of the prevalence of OsHV-1 in the week 3 challenge survivors in each immune conditioning treatment group was generated using a random sample of 20 surviving oysters. The prevalence was calculated as the number of oysters in which OsHV-1 was detected divided by the total number of oysters sampled from that treatment group. In addition, 95% confidence intervals were calculated using the binom.logit function from the binom package. The difference between groups in regard to OsHV-1 prevalence in the survivors was determined using a logistic regression model with the glm function. The parameter estimate was exponentiated to calculate the odds ratio for the detection of OsHV-1 between the reference group (sterile ASW) and each treatment group. The odds ratio for the reference groups was set at 1. The outcome for the model was the detection of OsHV-1 in the surviving oysters from each treatment group and the sole explanatory variable was the treatment group; the tank number of each oyster was included as a random effect.

## 3. Results

### 3.1. Immune Priming

In the 21 days after immune priming there was 12.5% (95% CI: 8.2–18.6) cumulative mortality in oysters injected with infectious OsHV-1 at 18 °C. The average OsHV-1 DNA concentration in oyster tissues at the time of death was 4.58 × 10^3^ (95% CI: 1.83 × 10^3^–1.14 × 10^4^) genome copies.mg^−1^. There was no mortality in the first 21 days following immune priming in any of the other treatment groups.

### 3.2. OsHV-1 Challenge

**Figure 2:**
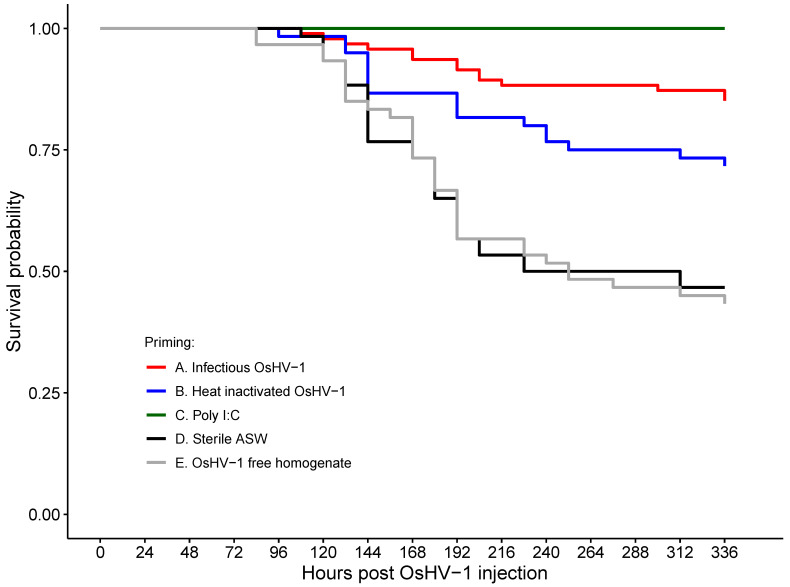
Kaplan–Meier survival curve after the OsHV-1 challenge, which occurred 3 weeks after immune priming.

High mortality occurred in both immune-priming-negative control groups: 53% in oysters injected with sterile ASW and 55% in oysters injected with the OsHV-1-free oyster tissue homogenate (Figure 2, Table 1). In contrast, the total cumulative mortality was 26.7% in oysters primed with heat-inactivated OsHV-1 and 12.8% in oysters primed with infectious OsHV-1 (Table 1), both corresponding to significant protection against mortality compared to the ASW control with a hazard ratio (HR) of 0.41 and 0.18 (Figure 2, Table 2) and a relative percent survival of 51.5% and 76.8%, respectively. There was no mortality after the OsHV-1 challenge in the group primed with poly I:C, indicating a strong protective effect compared to the ASW control (HR: 0.02) (Table 2). The OsHV-1-free oyster tissue homogenate did not have any protective effect compared to injection with ASW (HR: 1.03) (Table 2). There was no mortality in the negative control group in which oysters were challenged with the OsHV-1-free tissue homogenate.

**Figure 3:**
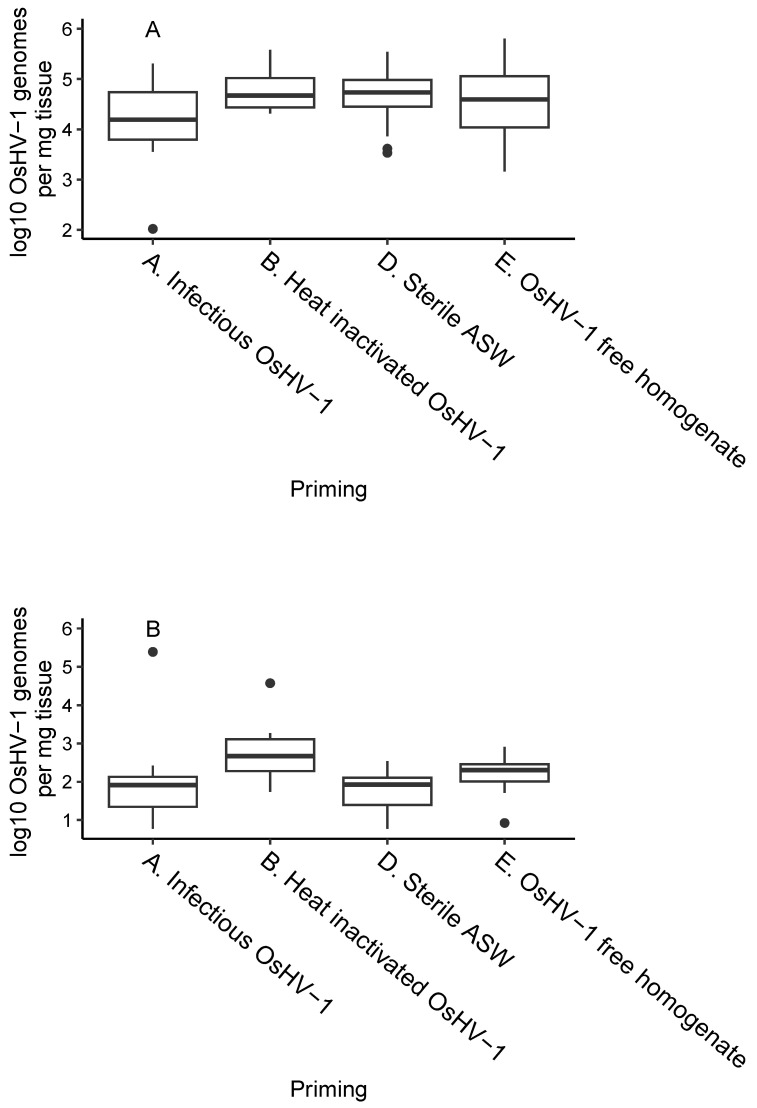
OsHV-1 DNA concentration in oysters (**A**) at the time of death and (**B**) in surviving oysters 14 days post challenge (Day 35). The oysters primed with poly I:C are not displayed here as they all survived to day 35 and OsHV-1 DNA was not detected in any of them. The box is bounded with the 1st and 3rd quartile and the black line indicates the mean; whiskers extend to the maximum and minimum except for data greater than 1.5 times the interquartile range from the mean, which are indicated by a point.

There was no significant difference in OsHV-1 DNA concentration between treatment groups in oysters that died following the challenge with OsHV-1 (*p* > 0.05), with concentrations of OsHV-1 DNA exceeding 10^4^ genome copies.mg^−1^ of oyster tissue (Figure 3) (Table 3).

Amongst the survivors at the end of the trial, OsHV-1 DNA concentrations were very low, being less than 10^2^ genome copies.mg^−1^ of oyster tissue, and the only oysters with a significantly lower concentration of OsHV-1 DNA in their tissues than those primed with sterile ASW were those primed with poly I:C (*p* < 0.001) (Table 3) in which OsHV-1 DNA was not detected.

At the conclusion of the trial, the prevalence of OsHV-1 in the survivors of the OsHV-1 challenge was highest in the group primed with sterile ASW (70%) and lowest in the group primed with poly I:C (0%) (Table 1). The latter group was the only one with a significantly lower prevalence of OsHV-1 compared to the group primed with sterile ASW. The odds of a surviving oyster in the group primed with poly I:C testing positive for OsHV-1 at the end of the trial was 0.02 that of the controls primed with ASW (Table 4).

## 4. Discussion

In previous experiments it was shown that prior exposure of *C. gigas* to OsHV-1, either in nature or in the laboratory, conferred some protection in a later challenge with a lethal dose of virulent OsHV-1 in the laboratory [22,33]. This also explains why the survivors of mass mortality events on oyster farms tend to be resistant during subsequent outbreaks [32]. In the present study, exposure to either infectious OsHV-1 or heat-inactivated OsHV-1 conferred protection. That heat-inactivated OsHV-1 produced a strong protective effect against subsequent exposure to virulent OsHV-1 confirms that immune priming with OsHV-1 does not require viral replication in the host. Experiments conducted more recently in a *C. gigas* hemocyte model enabled the same conclusion that viral replication was not required for an antiviral immune response [42]. This provides some information about the mechanisms of protection (see below). The current study also confirms an earlier observation that exposure to virulent OsHV-1 at a temperature non-permissive for mass mortality in Australia reduces the chance of mortality during a second challenge with virulent OsHV-1 at a permissive temperature [22,43]. The immune-priming-negative control oysters which were injected with sterile ASW experienced similar mortality when challenged with OsHV-1 at 22 °C compared to previous laboratory experiments at this water temperature. This illustrates the robust nature of this experimental infection model with enhanced repeatability through use of a cryopreserved inoculum [37,43,44,45].

A strong protective effect was observed with poly I:C, which was used as a reference immunostimulant in this experiment based on the prior literature [14,29]. Poly I:C is recognized by highly conserved intracellular TLRs as a pathogen-associated molecular pattern (PAMP) [46]. It is assumed that the mode of action is through excitation of the TLR3 receptor which binds to dsRNA and induces an antiviral immune response [14]. TLR3 expression also affects glucose homeostasis; therefore, the role of TLR3 is more complex than PAMP recognition alone [47]. Hemocytes of *C. gigas* have been identified as effector cells with increased reactive oxygen species production and expression of immune-related genes after poly I:C stimulation [42]. Poly I:C treatment influences immune gene expression and apoptosis in a sustained manner [48].

The level of protection against mortality due to OsHV-1 afforded by immune conditioning with poly I:C is dependent on the dose. Protection has been achieved previously with doses per oyster of 250 µg [14], 500 µg [13], 19 µg and 1.9 µg (50% survival) [29]. In the current study, the dose of poly I:C used for each oyster was 250 µg. The differences in protection afforded by injection of poly I:C, infectious OsHV-1 and heat-inactivated OsHV-1 observed in the current study may be due, in part, to the different quantities of nucleic acid injected or produced by each treatment. The complete absence or a low level of viral dsRNA in oysters conditioned with infective OsHV-1 may explain the lower level of protection against mortality in that treatment compared to poly I:C. If OsHV-1 behaves like other large dsDNA viruses, dsRNA could be produced during replication and so could stimulate similar pathways to poly I:C [49,50]. However, dsRNA is only a byproduct during replication, and would be present in a very low concentration if at all [49]. TLR was up-regulated in oysters injected with poly I:C but not in oysters infected with OsHV-1 [14,28,51,52], suggesting there may be little viral dsRNA present during replication of OsHV-1. Because non-replicating OsHV-1 (i.e., the heat-inactivated OsHV-1 treatment) also induced protection, pathways other than dsRNA binding to TLR3 may be involved in the anti-viral immunity observed in this treatment group.

OsHV-1 produces mRNA that is targeted by RNAi and has capsid and envelope proteins which are PAMPs that may stimulate different receptors to dsRNA [28,53]. Heating at 50 °C for 5 min was an effective method of disinfection of OsHV-1 infectivity, confirmed in a bioassay [37]. Viral proteins remain recognizable by host immune receptors after heating for 30 min at 56 °C, allowing heat-inactivated *Human alphaherpesvirus 1* (HSV) to adsorb to host cells and initiate a response [54]. The protective effect observed in this study from heat-inactivated OsHV-1 indicated that a relatively small dose of viral dsDNA and/or capsid and envelope proteins from the virus provided sufficient immune stimulation to result in a significant protective effect. The amount of viral dsDNA was likely to be less than 1 ng per oyster based on the OsHV-1 genome length and a dose of 10^6^ OsHV-1 genomes. Investigation of the immune pathways activated in oysters by exogenous nucleic acids and other PAMPs is required to differentiate the mechanisms of the protective effect afforded by the different treatments. Water temperature may also interact with and alter the response to immune stimulation in *C. gigas* and needs further evaluation [43,55,56].

Viral replication may be required for some types of immune priming, for example in mechanisms that require mRNA, such as RNAi. This requires pathogen mRNA to be cleaved and used as guide RNA in an RNA-induced silencing complex, leading to targeted cleavage of the viral mRNA [20,25,53,57]. The increased protection afforded by priming with live OsHV-1 compared to heat-inactivated OsHV-1, i.e., with a replicating infection, suggests that such a process may be involved.

Oysters which are in good condition and use a larger proportion of their energy reserves during the period of exposure to OsHV-1 have greater survival, possibly due to their ability to mount a more effective immune response than oysters in poor condition [9,58,59]. It will be important to understand the increased physiological demand imposed by an increase in antiviral immune activity following immune priming and whether this could affect the oysters’ growth rate and condition in order to avoid an unintended impact on oyster production. To produce a protective response upon encountering a pathogen for the second time, mollusks require mechanisms for immune memory [20]. Invoking a specific response which allows the immune system to differentiate between pathogens may have a lower physiological cost than general immunostimulation but would require using an immune stimulant with specific characteristics of the pathogen to produce an immune memory [23].

The application of immune conditioning or priming of Pacific oysters as a disease mitigation measure in aquaculture has several practical limitations. The conditioning method presented here requires intramuscular injection of each oyster, which is a problem given the high level of commercial production (11,345 tons in 2015–2016) [60]. Therefore, alternative delivery mechanisms are needed and immersion should be investigated as the most logical approach. OsHV-1 attaches to particles in plankton and is probably ingested during filter feeding; therefore, it could be delivered to spat in feed [61,62]. Transgenerational immune priming with poly I:C has been suggested as a viable method of efficiently conditioning oysters if the effect lasts for the entire production cycle because only broodstock need to be injected [13]. However, the use of poly I:C in commercial oysters used for food is unlikely to be acceptable due to the risk of toxicity. In mammals, the toxic effects of poly I:C include fever, hypotension, depressed total blood count and anemia, weight loss, focal hemorrhages and congestion [63,64,65,66]. Mortality followed a single 30 mg/kg dose of poly I:C in rhesus monkeys and 5 mg/kg in beagles [64,65]. The use of poly I:C would require research on the therapeutic index, residues in oyster tissues and withholding periods, as these are often required when chemicals are used in food-producing animals. Testing for residues would also be required as part of a food quality assurance program. For these reasons, conditioning with live or heat-inactivated OsHV-1 or its sub-components is potentially more acceptable to regulatory authorities than priming with poly I:C. While infectious OsHV-1 may be acceptable as an immune priming agent in regions where the disease is endemic, the use of heat-inactivated OsHV-1 rather than infectious OsHV-1 has the advantage of reducing the risk of initial mortality due to viable OsHV-1, even at a low water temperature [22]. Furthermore, using infectious OsHV-1 for an immune conditioning treatment introduces a biosecurity risk due to the potential to move infected oysters into an OsHV-1-free area. It would also complicate the interpretation of the results of disease surveys because oysters can test positive for OsHV-1 for nine weeks after a laboratory OsHV-1 challenge [22].

## 5. Conclusions

Oysters can be conditioned for increased survival when challenged with virulent OsHV-1 through prior exposure to OsHV-1 or by administering non-specific immune priming chemicals like poly I:C. There was no mortality associated with immune priming by injection of heat-inactivated OsHV-1, and with the development of more efficient administration techniques based on immersion, this could be used for reducing mortality caused by OsHV-1. Transgenerational impacts of immune priming with inactivated OsHV-1 should be investigated because they have been reported for poly I:C and could simplify large-scale application, requiring intramuscular injection only of broodstock. Further evaluation of priming with inactivated OsHV-1 under field conditions is warranted to assess efficacy. Controlled exposure to heat-inactivated or infectious OsHV-1 in OsHV-1 endemic areas would provide a platform for evaluation of the efficacy of the techniques during an outbreak. The specificity of the protective effect from immune priming requires further evaluation using other potential pathogens in order to understand the molluscan immune system and the physiological cost of immune conditioning on the oysters’ growth rate. Targeted investigations of immune responses identified in transcriptional studies are needed to understand the differences in immune response and potential mechanisms for immune memory. In addition, the duration of the protective effect needs to be determined along with interactions with water temperature, which clearly plays an important role in the outcome of OsHV-1 infection.

## Figures and Tables

**Table 1:** Total cumulative mortality and prevalence of OsHV-1 DNA in surviving oysters at day 35. The OsHV-1 challenge was administered 3 weeks after immune priming.

Challenge	Treatment Group	N	Mortality % (95% CI)	Prevalence % (95% CI)
Negative control	Heat-inactivated OsHV-1	20	0 (0–16.8)	-
	OsHV-1-free homogenate	20	0 (0–16.8)	-
	Infectious OsHV-1	30	0 (0–11.6)	-
	Poly I:C	20	0 (0–16.8)	-
	Sterile ASW	20	0 (0–16.8)	-
OsHV-1	Heat-inactivated OsHV-1	60	26.7 (17.0–39.2)	55.56 (33.0–76.0)
	OsHV-1-free homogenate	60	55.0 (42.4–67.0)	55.00 (33.6–74.7)
	Infectious OsHV-1	94	12.77 (7.4–21.2)	55.56 (33.0–76.0)
	Poly I:C	60	0 (0–6.0)	0.00 (0.0–16.8) *
	Sterile ASW	60	53.33 (40.8–65.5)	70.0 (47.3–85.9)

*p < 0.05 compared to other groups.

**Table 2:** Cox proportional hazards model describing the mortality in the 14 days after the challenge with OsHV-1 in oysters that had received immune priming treatments 3 weeks earlier. The group primed with sterile artificial sea water (ASW) was used as the reference group. The negative control group was not included in the model.

Treatment Group	Coefficient	Hazard Ratio (95% CI)
Sterile ASW	-	1
Heat-inactivated OsHV-1	−0.895	0.41 (0.25–0.67)
OsHV-1-free homogenate	0.025	1.03 (0.66–1.59)
Poly I:C	−3.802	0.02 (0.00–0.11)
Infectious OsHV-1	−1.699	0.18 (0.11–0.32)

**Table 3:** The estimated concentration of OsHV-1 DNA in oyster tissues at the time of death or 10 days post challenge in oysters that survived challenge with OsHV-1. Data were analyzed using a generalized linear mixed model. The outcome was log_10_-transformed viral concentration and the immune priming treatments were predictors, with tank included as a random effect. The estimate for the intercept variable for each model is the mean log_10_-transformed viral concentration for the reference group (conditioned with sterile ASW) and the estimate for the other groups is the difference between the mean of that group and the reference group.

OsHV-1 Challenge Outcome	Treatment Group (Parameter)	OsHV-1 Genomes.mg^−1^	Estimate (95% CI)	Std. Error	*p*-Value
Survivor	Sterile ASW (Intercept)	17.91	1.253 (0.76–1.75)	0.253	<0.001
	Heat-inactivated OsHV-1	24.32	0.133 (−0.61–0.88)	0.379	0.726
	OsHV-1-free homogenate	16.03	−0.048 (−0.75–0.65)	0.357	0.892
	Infectious OsHV-1	13.8	−0.113 (−0.83–0.61)	0.367	0.759
	Poly I:C	1	−1.253 (−1.95–−0.55)	0.357	<0.001
Mortality	Sterile ASW (Intercept)	4.48 × 10^4^	4.651 (4.33–4.97)	0.162	<0.001
	Heat-inactivated OsHV-1	2.89 × 10^4^	−0.19 (−0.73–0.35)	0.277	0.495
	OsHV-1-free homogenate	3.54 × 10^4^	−0.102 (−0.54–0.33)	0.221	0.645
	Infectious OsHV-1	1.30 × 10^4^	−0.536 (−1.12–0.05)	0.301	0.079

**Table 4:** Odds ratios for the presence of OsHV-1 DNA in oysters that survived a challenge with OsHV-1 3 weeks after different immune priming treatments. Data are from a generalized linear model with OsHV-1 DNA detection as the outcome and the immune priming treatment as the sole predictor, with tank included as a random effect to account for clustering.

Treatment Group (Parameter)	Estimate	Std. Error	*p*-Value	Odds Ratio (95% CI)
Sterile ASW (Reference)	0.8473	0.488	0.082	1
Heat-inactivated OsHV-1	−0.6242	0.6805	0.359	0.54 (0.14–2.02)
OsHV-1-free homogenate	−0.6466	0.6634	0.33	0.52 (0.14–1.9)
Infectious OsHV-1	−0.6242	0.6805	0.359	0.54 (0.14–2.02)
Poly I:C	−3.7917	1.1361	<0.001	0.02 (0–0.15)

## Data Availability

Original data are available upon reasonable request to the corresponding author.
